# Latissimus Dorsi Musculocutaneous Flap Inset Innovation in Breast Reconstruction 

**DOI:** 10.29252/wjps.8.3.394

**Published:** 2019-09

**Authors:** Ezatollah Rezaei, Kamrooz Pouryousef, Mohammad Karimi, Saeedeh Hajebi Khaniki, Ehsan Baradaran Sirjani

**Affiliations:** 1 Department of Plastic Surgery, Endoscopic and Minimally Invasive Surgery Research Center, Mashhad University of Medical Sciences, Mashhad, Iran;; 2 Student Research Committee, Mashhad University of Medical Sciences, Mashhad, Iran;; 3 Department of Biostatistics, Social Determinants of Health Research Center, Mashhad University of Medical Sciences, Mashhad, Iran;; 4 Department of Statistics, Mashhad Branch, Islamic Azad University, Mashhad, Iran

**Keywords:** Latissimus dorsi, Breast, Reconstruction, Implant

## Abstract

**BACKGROUND:**

Breast reconstruction is distinct among plastic surgery techniques. This study analysed the results of breast reconstruction with the Latissimus dorsi (LD) myocutaneous flap as a strategy for better coverage and positioning of the implant.

**METHODS:**

Twenty patients who underwent surgery between September 2013 and September 2016 were enrolled. Fourteen patients underwent reconstruction with LD and tissue expander (TE) exchanged later with implant. Six patients were reconstructed with LD and implant. The complications, problems, and aesthetic improvement associated with the use of implants placed under LD muscle were assessed.

**RESULTS:**

0ne case required an expander removal because of deflation of TE, also one case had seroma formation due to recurrence of breast cancer and also one case had seroma in donor site. No asymmetry was detected in the inframammary fold (IMF) position between reconstructed and normal regions. After the procedure, 80% of the patients reported that their expectations were met, 95% reported no functional limitations, and 5% reported mild limitations that ameliorated with physiotherapy. The placement of implants (prostheses or expanders) under the muscle with using the LD muscle flap to cover the implant improved the breast contour by softening the inframammary crease and positioning the implants in the upper and medial quadrants of the new breasts.

**CONCLUSION:**

Breast reconstruction using silicone implants and the LD muscle flap can have excellent outcomes with low rates of complications. Placing the implant under a layer of muscle improved the harmony of the upper quadrants during breast reconstruction.

## INTRODUCTION

Breast cancer is the most common female cancer in western countries,^1^^,^^2^ and annually about 8000 new cases are diagnosed with breast cancer in Iran.^3^ Breast reconstruction was performed primary and delayed. Immediate breast reconstruction (IBR) was performed at early stage breast cancer (DCIS and T1-2 tumors), while those with presumptive radiotherapy or local advanced breast cancer (LABC) are recommended delayed procedures^4^^,^^5^ that should be performed two years after adjuvant therapy due to highest risk of recurrence in this period. Implant-based reconstructions has been developed by several autologous methods.^6^^,^^7^

An autologous technique is transferring of tissue (as a pedicled flap or free flap with microsurgical technique) to the site of mastectomy in chest wall. In general, autologous breast reconstruction is more demanding than implant-based reconstruction.^6^^,^^7^ Latissimus dorsi (LD) flap was a commonly used reconstructive technique in 1970s.^8^^-^^10^ Sometimes, it is combined with an implant to achieve the desired volume and to be used alone as an extended flap, with fatty tissue harvested together with the muscular tissue.^11^ The lateral thoracodorsal flap (“Göteborg” flap) is added from the lateral-dorsal thoracic wall to form the lateral part of the breast that is frequently combined with implants.^12^

The transverse rectus abdominis myocutaneous flap (TRAM) was introduced for breast reconstruction in 1980s; that can be used either as pedicled or a free flap.^13^^-^^15^ Today, the free TRAM is often replaced by perforator flaps, such as the deep inferior epigastric perforator (DIEP) flap or the superficial inferior epigastric artery (SIEA) flap.^16^^-^^19^ Other flaps include the free gluteal flap,^20^ the free anterolateral thigh flap^21^ and the free transverse musculocutaneous gracilis (TMG) flap.^22^ The choice of a reconstructive method is a multifactorial and depend on oncological considerations, local traditions, and the patient’s condition and preferences.

## MATERIALS AND METHODS

In this study, we identified all patients who underwent tissue expander (TE) implant breast reconstruction at Ghaem Hospital, Mashad, Iran between September 2013 and September 2016. An extensive retrospective review medical record of patients was performed in order to screen possible study inclusion and collect demographic, therapeutic, and operative data for subsequent analysis. Totally, 26 patients were identified as having total mastectomy surgery with or without radiotherapy after mastectomy at Ghaem Hospital. 

**Fig. 1:**
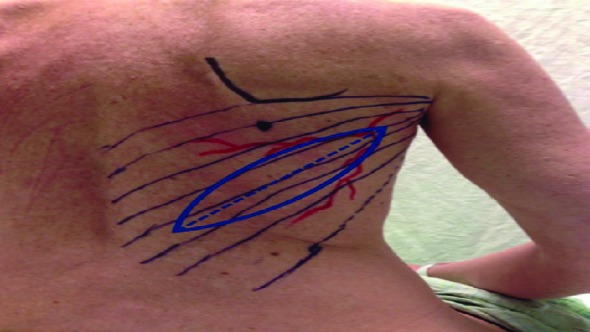
Preoperative markings

Six of those patients were excluded because they chose to undergo autologous reconstruction with TRAM. After exclusions, a total of twenty eligible patients were identified with twenty breasts that underwent completed LD and TE implant reconstruction. All complications requiring additional surgery or hospitalization were recorded. For the purposes of this study, we defined reconstruction failure as removal of the permanent implant following initial successful expander-implant exchange. Length of follow up was six months. Posterior markings were performed with oblique skin islands orientations (Figure 1).

In lateral decubitus position after prepare and drape, the procedure was begun by incising the skin island and dissecting through the subcutaneous tissue to the muscle. Then muscle flap was released and elevated completely from its origin, and the pedicle was isolated. After a subaxillary tunnel creating, the flap was positioned on the anterior chest by gently pushing it through the tunnel. Inseting of the transferred skin flap into its proposed position was undertaken to ensure adequate reach without compromising the vascular pedicle. 

Once a satisfactory length and position was achieved, 2 suction-drain was inserted and the back incision was closed in 3 layers. Then patient was repositioned and draped in the supine position. In this position after incision on previous scar of mastectomy and deletion of the scar, skin and subcutaneous tissues were dissected (upper and lower flap) around the incision. This dissection was limited to secure a permanent space for the implant, which prevented excessive movement of the implant too. Then washing and haemostasis of new pocket were completed. When an acceptable position was attained, suturing of the lattissimus flap was done to the anterior skin pocket flap with non-absorbable sutures (nylon 2/0) as follows: 

Marking of three points in superior pole border over upper skin flap was conducted, mid-point was in midclavicular line and other points in 3-5 cm distance in medial and lateral of mid-point. One point was in medial border (2 cm lateral to midsternal line), and 3 points in the inframammary fold (IMF) line align with upper pole points in lower skin flap. Three horizontal mattress nylon 2/0 sutures were passed superiorly in free border of LD muscle with 3-5 cm distance between them. Three sutures in inferior border and one suture in medial border of LD muscle as same way as superior border was done and grasped.

A 1-2 mm cutaneous stab incision was made in marked points. A thin reverdin needle was introduced through the stab incision, the free end of suture passed reverdin, the suture with reverdin was then pulled out from skin flap, the suture was hold by grasper, and freed from reverdin needle. The reverdin needle was then introduced again through stab incision in different paths, the other end of suture was passed and pulled out from stab incision and grasped. In other marked points again and similarly, this technique was carried out.

The tissue expander was placed over pectoralis major and under LD muscle in manner that whole of LD muscle covered the tissue expander. In patients with radiation history, we placed the tissue expander under pectoralis major. At the same time in lateral of pocket, we fixed the LD muscle to chest wall in anterior axillary line with 2/0 nylon. This suture prevented migration of flap and implant and protected the pedicle from tension. The tissue expander port was placed in lateral chest wall far from the expander and incisions. After insertion of suction-drain, seven sutures were gently pulled and tied and deeply buried in subcutaneous tissue. Then the skin island was closed in two layers and then the expander was filled with 100-150 mL of saline.

## RESULTS

A total of 20 consecutive women who were diagnosed with breast cancer and had undergone total mastectomy prior to reconstruction underwent LD musculocutaneous flap delayed breast reconstructions. Characteristics of the patients were summarized in Table 1. Most of the patients (65%) underwent breast irradiation, and the median time from mastectomy to breast reconstruction was 31 months. Fourteen patients underwent two-stage breast reconstruction with the initial TE insertion replaced by a form stable cohesive gel anatomical implant. 

**Table 1:** Patients’ demographic and clinical characteristics

**Variable**	**Data**
Age, years	40.6±7
Body mass index, n (%)	
<25 kg/m^2^	10 (50)
25 kg/m^2^ to 29 kg/m^2^	9 (45)
≥30 kg/m^2^	1 (5)
History of radiation therapy, n (%)	13 (65)
History of chemotherapy, n (%)	19 (95)
LD skin paddle height (cm)	9.6±1.4
TE	Moderate height, base width
	12-14 cm, 20%-30% overfill
TE volume (mL)	575±35
Time interval between mastectomy and delayed breast reconstruction	35±9
(months)	
Time interval between tissue expander insertion and implant	8±3
exchange (months)	
Length of hospitalization (day)	4±1
Contra lateral surgery, n (%)	6
Duration of surgery	183±17
Removal of last drain	15±5

LD: Latissimus dorsi, TE: Tissue expander, Data were presented as mean±SD, unless otherwise indicated

**Fig. 2:**
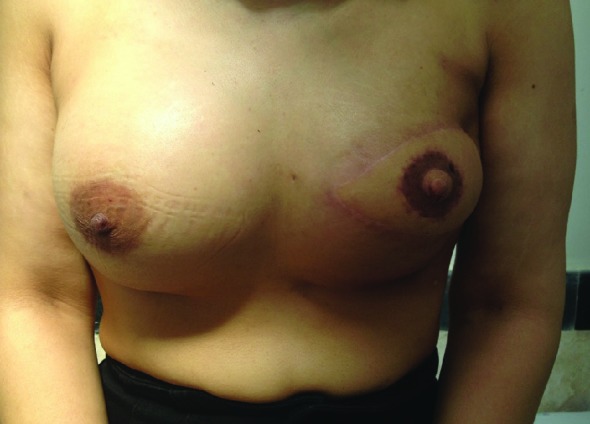
Post-mastectomy reconstruction after 6 months

Figure 2 shows a typical result following LD musculocutaneous flap breast reconstruction. The procedure including repositioning and redraping was completed in approximately 3.9 hours, ranging from 2.5 to 6 hours, and with increasing experience, the procedure was shortened to about 2.45 hours. No blood transfusion was required during surgery. Whereas, in 14/20 cases in whom the classic expander was used, exchanging of the expander for permanent implant was performed in 12 patients, and in 6 patients LD+implant in the first surgery. 

In one patient, the TE was deflated and TE was exited and breast reconstruction was done with only LD. So one patient was excluded from the study, due to recurrence of breast cancer and seroma formation. The length of hospital stay ranged from 3 to 6 days, with a median of 4 days. Postoperative complications are summarized in Table 2. One patient (5%) developed dehiscence of the overlying skin envelope. Dehiscence was managed either by debridement, allowing healing by secondary intention. This was achieved without disturbing the underlying prosthesis in the patients, and was followed by successful re-expansion of the breast mound. No patient developed LD flap loss. 

**Table 2:** Complications noted post-surgery

**Complication**	**Number **	**Radiation history**	**P value**
Wound dehiscence±HTS	1 (5%)	+	
TE deflation and extrusion	1 (5%)	+	0.52
Misplacement of port site	1 (5%)	+	
Limited arm movement	1 (5%)	+	
Seroma	1 (5 %)	+	
Total	5 (25%)	5 (13)	

TE: Tissue expander, HTS: Hypertrophic scarring

One patient (5%) required expander removal for late postoperative exposure and deflation 3 months after surgery. There had been no case with infection. Misplacement of the port site was encountered in one patient (5%), in whom the prosthesis was salvaged by reoperation and revision of port. Donor site complications were seen in 2 patients, one patient with seroma formation and one patient with limited arm movement were ameliorated with physiotherapy. During the follow-up period with a median of 6 months, there was no evidence of local or distant relapse. The patients’ aesthetic results were summarized in Table 3.

**Table 3:** Patients’ reported satisfaction

**Scoring by patients**	**Frequency**	**Contra lateral surgery**
Completely satisfied	13.18 (72.2%)	6.13
Moderately satisfied	3.18 (16.7%)	-
Poorly satisfied	2.18 (11.1%)	-

## DISCUSSION

The LD flap could be a safe method for breast reconstruction.^23^ It was shown in some studies that the flap would give enough volume for reconstructing of small to medium breasts and in some cases, if It is necessary, a lot of tissues would undergo extended LD flap procedure.^20^ On the other hand, prosthesis was used, but a proper decision of the size was difficult. Throughout a one-stage operation involving LD flap and an implant, an oversized skin paddle was employed to enhance the degree of the mastectomy skin envelope, thereby letting the foremost conceivable implant to be used for reconstruction. An oversized implant was important as a result of LD muscle atrophies during first year after operation and its final contribution to the volume of the breast was doubtful. 

However, though symmetry would be achieved with this method, it’s at the cost of extra scars that scale back patient satisfaction. Moreover, fat grafting was an alternative choice to obtain symmetry in single-stage LD reconstruction.^24^ However, this technique incorporated a modest result on tissue growth, and also the patient needed to be encouraged and willing to bear multiple fat attachment sessions. With the presence of an implant, safety was overriding, and that we liked small-volume fat injections employing a 16-gauge blunt-tip cannula.

In distinction, the two-stage approach took time for soft tissue healing and also the inevitable atrophy of the LD muscle before a choice was formed concerning the size of the ultimate implant. This can be followed by the second stage, six to twelve months later, within which the skin was step by step distended to reach a volume analogous the alternative side, followed by 20%-30% overexpansion to make a natural degree of ptosis. This technique eliminates the necessity for an oversized LD skin paddle externally and compassed the scars to the nipple-areolar complicated, leading a more robust aesthetic result. 

Any excess skin was de-epithelialized and used as a soft tissue to protect natural augmentation and to refine breast projection. What is more, it lessened the necessity for extended LD flaps, which were generally related to augmented donor site morbidity. Also this technique covered whole of tissue expander and decreased the chance of extrusion of tissue expander or implant in patients with history of radiotherapy. We experienced only one extrusion of tissue expander. Other choices for two-stage breast reconstruction involved employing a tissue expander covering with LD in lower pole.

In the first stage, the tissue expander was placed subpectorally, and its exposed lower pole was covered with LD. Fixation of LD within the superior pole was subdermal with absorbable suture and within the lower pole fixation was done in continuous manner.^25^ The steps of tissue growth and implant exchange were just like our technique, however in our technique we had a tendency to place the expander over pectoral muscle and whole of expander was underneath LD. Fixation was carried out separately with absorbable suture and reverdin needle. 

This approach might succeed symmetry, provide the skin envelope well hold and vascularized. Also time of surgery was reduced. In this study, time of surgery was 3.1 hours compared to minimum of 3.9 hours in different techniques. We believe that decrease in operational time and ease of our technique were the superb advantages. By employing a LD flap, operative complications, like implant extrusion, seroma, and infection reduced.^25^ In the current study, we have designed the skin paddle in oblique direction and that we were still able to harvest enough fat from the scapular and lumbar. The selection of the skin design was different, for example some researchers used the crosswise skin paddle.^26^^,^^27^ Others have opted the crescent-shaped paddle represented by Marshall *et al.*^28^ Dorsal flap necorsis could be a potential downside and it has been reported by many authors. The frequency of necrosis in the study of Chang *et al.* was 16% whereas, Delay *et al.* confirmed 3% incidence.^27^ In the current report, we had no dorsal flap necrosis. The first wound closure of the donor area ought to be tension free. The best width of the skin paddle was estimated, but generally varies between 7 and 9 cm. 

Based on the demand for overcorrection, reconstruction must be noted as the volume of flap during the follow up decreases up to 25% and ends the smallest volume within 12 months,^29^ resulting into muscle atrophy an the maintenance of the thoracodorsal nerve might facilitate the preserve of the muscle bulk.^29^ The overall patient satisfaction during this study was excellent, while 16 patients out of 20 were completely or moderately satisfied. The disadvantage of the LD flap was its donor site morbidity. Noticeably, this included a high incidence of seroma ranging from 9% to 19% in some studies and up to seventy nine percent among the others.^29^

In this study, the incidence of seroma was 5% (1/20). Another problem of LD was the contour deficiency on one side of the back.^19^^,^^29^ In the current study, this deformity was not observed among most of patients. Only in two patients, it was minimal and acceptable. Regarding shoulder function, the functional deficit was usually low and acceptable. In our study, only one patient experienced mild limitation in shoulder movement that resolved with physiotherapy. The flap was primarily indicated for those who were not suitable candidates for TRAM flaps or for that group of patients, who would prefer the back donor site and are reluctant to proceed for the prolonged recovery of the pedicled TRAM or for the possible morbidity and the complexity of free tissue transfers. 

One of the disadvantages of the flap was the high incidence of seroma. The rest was the restriction in the size of the flap which made it inappropriate for some patients with breast ptosis unless a contra lateral procedure has been done. In the absence of contraindications, immediate breast reconstruction with implants can be offered to and performed on patients with invasive breast cancer without any negative effect on oncological safety. Low-cost plastic cast measurement gave more exact values for breast volume than various methods for 3-D measurement. 

However, more advanced 3-D technology may prove to be an important and useful technique for the objective evaluation of reconstructive breast surgery. Shape can be measured objectively by two-dimensional technique based on 3-D laser scanning. Moreover, the round permanent expander method failed to serve as a one-stage procedure, mostly due to upper pole fullness and lack of ptosis. The crescent two-stage expander method gave a more natural shape of the breast; both the patients and the panels of experts and people scored the highest regarding the shape and symmetry. Quality of life significantly improved 1.5 years postoperatively in both groups, with no major differences between the groups.

## CONFLICT OF INTEREST

The authors declare no conflict of interest.
